# Modeling the public health impact of different meningococcal vaccination strategies with 4CMenB and MenACWY versus the current toddler MenACWY National Immunization Program in Chile

**DOI:** 10.1080/21645515.2021.1996808

**Published:** 2021-12-10

**Authors:** María Gabriela Graña, Gabriel Cavada, Marjorie Vasquez, Jing Shen, Johan Maervoet, Johan Klint, Jorge A. Gómez

**Affiliations:** a GSK, Santiago, Chile; b Universidad de Chile, Santiago, Chile; c GSK, Wavre, Belgium; d Parexel, Wavre, Belgium; e Parexel International, Stockholm, Sweden; f GSK, Buenos Aires, Argentina

**Keywords:** Chile, invasive meningococcal disease, epidemiology, vaccination, 4CMenB, MenACWY, dynamic model

## Abstract

Invasive meningococcal disease (IMD) is an uncommon yet unpredictable, severe, and life-threatening disease with the highest burden in young children. In Chile, most IMD is caused by meningococcal serogroup B (MenB) and W (MenW) infection. In response to a MenW outbreak in 2012, a toddler vaccination program was implemented using quadrivalent meningococcal conjugate vaccine against serogroups A, C, W and Y (MenACWY). The vaccine program, however, does not protect infants or other unvaccinated age groups and does not protect against MenB IMD. Since 2017, MenB IMD cases are becoming increasingly prevalent. Using a dynamic transmission model adapted for Chile, this analysis assessed the public health impact (reduction in IMD cases, long-term sequelae, deaths, and quality-adjusted life-years) of six alternative vaccination strategies using MenACWY and/or the four-component MenB (4CMenB) vaccine in infants, toddlers, and/or adolescents compared to the National Immunization Program (NIP) implemented in 2014. Strategies that added infant 4CMenB to MenACWY in toddlers or adolescents would prevent more IMD than the current NIP, observed within the first 5 years of the program. Replacing the NIP by an adolescent MenACWY strategy would prevent more IMD in the longer term, once herd immunity is established to protect unvaccinated infants or older age groups. The strategy that maximized reduction of IMD cases and associated sequelae in all age groups with immediate plus long-term benefits included infant 4CMenB and MenACWY in both toddlers and adolescents. This analysis can help policymakers determine the best strategy to control IMD in Chile and improve public health. A set of audio slides linked to this manuscript can be found at https://doi.org/10.6084/m9.figshare.16837543.

## Plain Language Summary (PLS)

What is the context?

Invasive meningococcal disease (IMD) is a severe, sometimes fatal, unpredictable disease with highest rates in infants, young children, and adolescents. It is caused by different serogroups of *Neisseria meningitidis* bacteria. Most cases in Chile are due to meningococcal serogroups B (MenB) and W (MenW). Following a MenW IMD outbreak in 2012, vaccination was introduced, leading to the current National Immunization Program (NIP) in toddlers with quadrivalent meningococcal conjugate vaccine (MenACWY) (protecting against IMD caused by MenA, C, W, and Y).

What is new?

A disease model to predict the impact of vaccination strategies in the Chilean population compared six alternative strategies, using the multi-component MenB (4CMenB) vaccine for infants (protecting against MenB, with potential cross-protection against MenW and Y IMD) and/or the MenACWY vaccine for toddlers and/or adolescents.

What is the impact?

Results, compared to the NIP, show that: Strategy 1 (a program targeting only infants with 4CMenB) would reduce more MenB cases but fewer MenA, C, W and Y cases resulting in a lower reduction of total IMD cases in the long term; Strategy 3 (a program targeting only adolescents with MenACWY) would have a similar effect to the NIP in the short term but a far greater IMD reduction in the long term (as vaccinating this age group eventually reduces transmission to other age groups, reducing their risk of disease); all the other strategies targeted more than one age group, further reducing numbers of IMD cases compared with the NIP. The greatest benefits were seen with infant 4CMenB vaccination combined with toddler and adolescent MenACWY vaccination. Results can help policymakers determine the best IMD strategy to maximize the benefits of available meningococcal vaccines.

## Introduction

Invasive meningococcal disease (IMD), which typically manifests as meningitis or sepsis, is a severe and life-threatening disease.^1^The disease is infectious, and its epidemiology is unpredictable,^2^ with outbreaks causing a major public health burden. IMD occurs mostly in infants and young children, with a second peak in adolescents in some countries.^1,3^ The highest burden of disease tends to be in infants and young children, while adolescents tend to have the highest transmission rates.^1,4,5^

IMD is associated with an important risk of mortality within days of infection (up to 15% in treated cases and up to 80% in untreated cases^1^), and long-term physical, neurologic and psychologic sequelae can occur in up to 20% of those surviving the acute phase of the disease^1,3,6^ or up to 51.6% among infant survivors.^7^ In Chile, the case-fatality rate (CFR) has been steadily increasing from around 10% before 2010 to 28% (2012–2015) and peaking around 32% (in 2019).^8^ In addition to the consequences on the health of patients, IMD has a high emotional and economic impact on families and also a long-term social impact.^9^

In Latin America, five meningococcal serogroups (MenA, MenB, MenC, MenW, and MenY) cause most endemic and epidemic diseases with variations reported by country and age.^2,3^ Typically, most cases are caused by MenB and MenC with an increase in MenW associated with the clonal complex sequence type 11 reported in Argentina and Chile.^10^ Vaccination remains the most effective means of preventing IMD.^1^ Various vaccines and strategies are used worldwide, i.e., conjugate vaccines are more immunogenic than previous polysaccharide vaccines,^11^ and include quadrivalent vaccines targeting MenACWY IMD with capsular polysaccharides conjugated to a carrier protein, and monovalent conjugate vaccines targeting MenC or MenA. Recombinant protein vaccines are available targeting MenB.^11,12^ Vaccination with the four-component MenB vaccine (4CMenB) (Bexsero, GSK) and the quadrivalent meningococcal conjugate vaccine (MenACWY) (Menveo, GSK) provides protection against IMD caused by these common serogroups. 4CMenB can be administered from the age of 2 months (with a flexible 2- or 3-dose schedule^13,14^) and provides direct protection against MenB IMD, with recent evidence showing that 4CMenB has the potential to cross-protect against MenW and MenY IMD.^15–17^ A 4CMenB vaccination program in infants could therefore directly reduce the burden of IMD in the age group with the highest incidence. MenACWY can be administered to toddlers or adolescents (with one dose from the age of 2 years^18^) and is also approved in Chile for infants (from 2 months old, flexible 2- or 3-dose schedule^19^) and provides direct protection against MenACWY IMD. It also protects against asymptomatic carriage of MenCWY, thereby indirectly reducing transmission.^20^ A MenACWY vaccination program in adolescents could therefore reduce transmission in the group with the highest carriage rates,^5^ providing a means to potentially induce strong herd protection in the whole population.

In Chile, MenB IMD was predominant until 2012, and there was a lower but rising incidence of MenW IMD since 2011. Between 2012 and 2014, an outbreak of MenW IMD occurred, with the largest groups affected being children under 5 years of age (46.7% of cases), adults aged 20–60 years (28.3%), and adults over 60 years of age (13.3%).^3,21,22^ The rapid increase in MenW cases, representing 58.0% of IMD cases by 2012 and 75% by 2014,^3^ was associated with higher mortality rates than previously reported with IMD cases,^3,23^ i.e., CFRs were around 15.0% in 2011^22,24^ and reached 28% in 2012 and 2015.^23^ In 2012, 10% of patients developed severe sequelae (e.g., limb amputations, hearing loss, and neurologic damage).^21^ As a response to this outbreak, the “W-135 Action Plan” was initiated in 2012 – a catch-up vaccination campaign with MenACWY in children aged 9 months to 5 years, and in 2014, MenACWY was included in the National Immunization Program (NIP), administered as a single dose at the age of 12 months.^22,23^ In addition to the dose in toddlers, the NIP offers MenACWY since 2017 and 4CMenB since 2019 for at-risk populations.^25,26^

The Chilean strategy was effective in reducing the incidence and mortality of MenW cases in the targeted age groups.^3,23^ This single-dose toddler prevention strategy remains in place today. IMD incidence in the overall population declined following vaccination (from 0.8 in 2012–2014 to 0.3 in 2017 per 100,000),^3^ but the decline of 92.3% in vaccinated age groups (under 5 years of age) between 2012 and 2016^23^ did not produce herd protection in unvaccinated age groups, possibly as no adolescents were vaccinated, and the second highest peak in incidence was observed in older adults.^23^ Cases due to MenW continued to dominate until 2017,^3^ but the proportion of IMD cases due to MenB are increasing since 2018. In 2019, MenB cases were predominant (46% overall) and especially among infants (67%).^8^ These changes highlight the need to reassess the current meningococcal vaccination strategy in Chile.

The objective of this study was to explore the potential public health benefits of six vaccination strategies with infant 4CMenB and toddler or adolescent MenACWY administered as single vaccines or in various combinations, compared to the current toddler MenACWY NIP in Chile.

## Materials and methods

**Figure 1.**
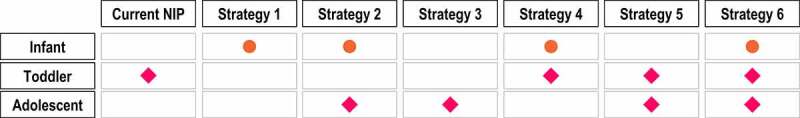
Six vaccination strategies modeled. Overview of the vaccines included for infants and/or toddlers and/or adolescents in each vaccination strategy modeled and in the current National Immunization Program (NIP).

A previously published Dynamic transmission-based Cost-Effectiveness (DyCE) model, developed to evaluate the impact of 4CMenB and MenACWY in England,^27,28^ was adapted to simulate meningococcal carriage transmission and IMD incidence in the Chilean population and to assess the potential impact of different vaccination strategies (Figure 1).

The DyCE model has two parts: 1) dynamic transmission, and 2) decision tree. In the dynamic transmission part, the model simulates transmission of meningococcal carriage over time and estimates the number of IMD cases (by MenB, MenACWY, or “MenOther” serogroups) with each vaccination strategy, compared to the current NIP. As an individual acquires carriage, an instantaneous risk is assumed that the individual develops IMD, determined by the age- and serogroup-specific case-carrier ratio. The model assesses direct prevention of disease in vaccinated individuals and reduced carriage acquisition with MenACWY vaccination, leading to indirect prevention from herd immunity. In the decision tree part, the model estimates the long-term consequences for each IMD case: the probability of death in the acute phase, of recovering, or developing a range of 16 types of sequelae in survivors and the health-related quality of life associated with each health state. The DyCE model assesses vaccine impact over time, assuming the new vaccination strategies are implemented from 2021 onwards.

The main health outcome is the percent reduction in IMD cases over time, compared to a scenario reflecting the current NIP in Chile, in the short (5 years) and longer term (25 years). Additional health outcomes include the number of cases of long-term sequelae among IMD survivors, IMD-related deaths in the acute phase, and quality-adjusted life-years (QALYs) lost.

### Key data for the model adaptation for Chile

**Figure 2.**
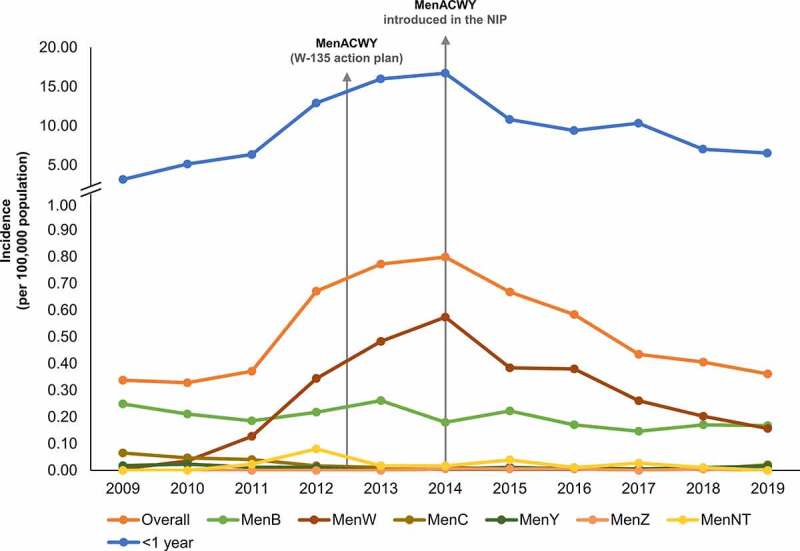
IMD incidence in Chile from 2009 to 2019 – by serogroup in the overall population, and in infants. IMD incidence can change unpredictably. The rapid increase in serogroup W (MenW) cases (from 2011) led to the introduction of MenACWY vaccination (from 2012 in the ‘W-135 Plan’ and from 2014 in the National Immunization Program [NIP]). IMD incidence rates are highest in infants. Abbreviations: MenACWY, quadrivalent meningococcal conjugate vaccine; MenB/MenW/MenC/MenY/MenZ/MenNT, serogroups B/W/C/Y/Z/NT IMD.

Demographic and IMD data from Chile were used to fit the model. Figure 2 highlights the unpredictable nature of IMD, showing the changes in IMD incidence by serogroup in Chile between 2009 and 2019, and the highest incidence in infants.

The starting population from 2013 (reported in the 2017 Census published by Instituto Nacional de Estadísticas^29^) was used, the year was chosen to coincide with the vaccination strategy in response to the MenW outbreak starting in 2012 and subsequent NIP introduced in 2014.^30^ Projections for the number of births over the model’s time horizon were based on birth data from 1990 to 2017,^31^ and age-specific mortality rates for all age groups (between 0 and 99 years) were obtained from life tables from the Chilean Department of Statistics and Information in Health.^32^

**Figure 3.**
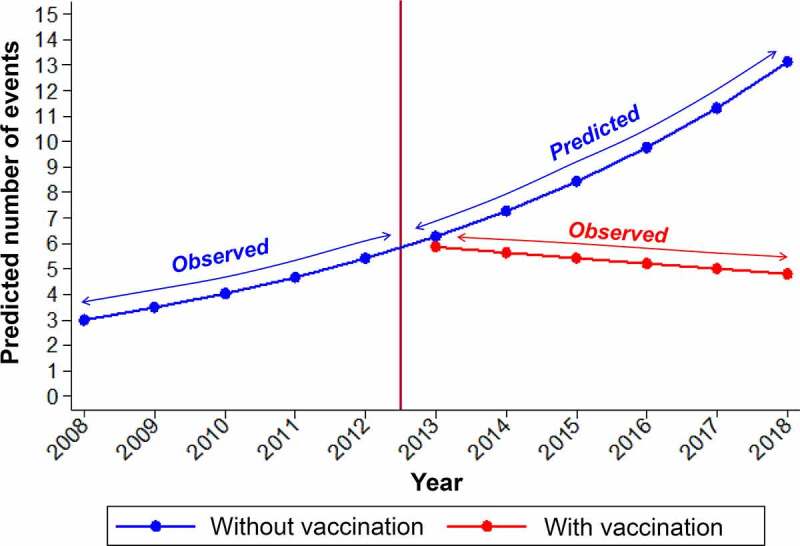
Predicted IMD cases (2013–2018) assuming no vaccination compared with observed cases (2013–2018) after vaccination programs were introduced in 2012. Prediction of invasive meningococcal disease (IMD) cases (2013–2018) based on observed cases (2008–2012) and assuming no vaccination program was introduced in 2012, compared with observed cases (2013–2018) following the implementation of vaccination strategies to control the outbreak.

IMD cases from 2008 to 2018 stratified by serogroup (A, B, C, W, Y, X, and ‘Other’) and 1-year age groups (for ages 0 to 99 years) were obtained from the Institute of Public Health in Chile, through Transparency Law.^33^ For infants (age <12 months), IMD incidence by month of age was used in the model to obtain more granular results. Model inputs for MenB and ‘MenOther’ were based on the observed IMD cases from 2008 to 2018. No cases of MenA and MenX were recorded. Model inputs for MenACWY IMD were based on observed IMD cases from 2008 to 2012, representing incidence data before any large-scale meningococcal vaccine programs were introduced in Chile. For the year 2013 and onwards, to reflect the increase in IMD cases due to the outbreak starting in 2012, the average number of MenACWY IMD cases was increased from the pre-outbreak average of 27 cases per year (years 2008–2012) to 119 cases per year. This estimate represents the average number of MenACWY IMD cases that would have occurred if no meningococcal vaccination programs were introduced. To derive this estimate, the evolution of the observed number of IMD cases from 2008 to 2012 (using Poisson regression modeling, in STATA software version 16 [StataCorp LLC, College Station, TX, USA]), was used to project the number of cases up to 2018, assuming that no vaccination had been implemented. The average projected estimates from 2013 to 2018 were compared with observed cases from the same period with vaccination programs in place, to calculate the reduction in the annual cumulative incidence of IMD. The temporal effect was expressed as an incidence rate ratio (IRR). The estimated projected scenario (without vaccination) was built considering an IRR = 1.16 (*p* = .003), while cases observed from 2013 to 2018 (with vaccination) remained constant: IRR = 0.96 (*p* = .355). That is, a reduction of 42.4% in annual cumulative incidence was estimated (Figure 3).

IMD mortality data in the acute phase were obtained from the Hospital Discharge Database 2012–2018 in Chile.^34^ CFR for each age group was estimated based on 61 deaths and 568 IMD cases registered (see Appendix Table 1 for CFR by age group).

**Table 1.** Vaccination parameters for 4CMenB and MenACWY

	4CMenB			MenACWY	
	Infant and toddler			Toddler	Adolescent
Dosing	3 m	5 m	12 m	12 m	11 y
Vaccine effectiveness (%)	0	80.0^39^	82.8^39^	79.0^40^	79.0^40^
Average duration of protection (months)	33^41^	33^41^	38^41^	48^42–44^	187^45^
Carriage effect (%)	0			36.2 against MenACWY IMD^20^	
Vaccine effectiveness potential cross-protection (%)	Calculated cross-protection against MenACWY IMD:			No cross-protection against MenB IMD	
	Infant (3 m, 5 m) – 78.9				
	Toddler (12 m) – 74.1				
	Calculations use: 80 MenW IMD, 93.8 MenY IMD, 0 MenAC IMD^17^				
Coverage (%)	99.0^a^	99.0^a^	96.0^b^	96.0^b^	89.9^c^

Infant is <12 months, Toddler is ≥12 months old; Adolescent is ≥11 years old. Vaccine effectiveness for 4CMenB based on available data at time of analysis. Conservative assumption of 0% effectiveness after Dose 1 assumed. Vaccine cross-protection for 4CMenB calculated assuming potential cross-protection against MenW and MenY only.

^a^Based on Chilean data for the hexavalent vaccine at similar ages.^38^

^b^Based on Chilean data for the measles, mumps, and rubella (MMR) vaccine at 12 months of age.^38^

^c^Based on Chilean data for the first dose of the human papillomavirus (HPV) vaccine at 11 years of age.^38^

Abbreviations: 4CMenB, four-component meningococcal serogroup B vaccine; IMD, invasive meningococcal disease; MenACWY, quadrivalent meningococcal conjugate vaccine; MenACWY/MenAC/MenW/MenY IMD, serogroups A, C, W, Y/A, C/W, or Y IMD; m, months; y, years.

The risk of developing long-term sequelae in survivors was not available for Chile and was based on the literature, along with the utility loss for each sequela (see Appendix Table 2).^28^ While observational studies suggest multiple IMD sequelae can occur,^6,9,35^ due to limited data, the model assumes that the probability of developing a particular sequela is independent of whether the patient develops any other sequelae and does not differ by age.

As limited age-specific carriage data for Chile were available (i.e., reports of 4% carriage in university students^36^ and 7.6% in adolescents aged 14 to 19 years,^24^ with no carriage data in infants), carriage prevalence by model age group was based on a systematic review by Christensen et al.,^5^ e.g., assumed to be 4.5% in infants and peaking in adolescents (23.7% in 19-year-olds). The distribution of serogroups by carriers was 17.71%, 6.29%, and 76.00% for MenB, MenCWY, and “MenOther,” respectively, based on the study by Soeters et al.^37^

### Vaccination parameters

The parameters relating to vaccine properties were obtained from the literature,^27,28^ and the proportions covered by vaccination were obtained from local data for Chile^38^ (Table 1).

4CMenB has been shown to provide direct protection against MenB IMD and cross-protection against other serogroups, such as MenW and MenY, from immunogenicity data^17^ and from real-world evidence following introduction of routine 4CMenB infant vaccination in the UK.^15^ The model used the 4CMenB human serum bactericidal assay (hSBA) results, showing that 80.0% of MenW and 93.8% of MenY strains are killed by 4CMenB antibodies,^17^ to approximate vaccine effectiveness against MenW and MenY. The overall cross-protection against MenACWY IMD in the model was calculated using an IMD incidence-based weighted approach, as in other meningococcal disease transmission models.^46^ Conservatively assuming 0% effectiveness against MenA and MenC IMD (due to low incidence in Chile [Figure 2]), the calculated cross-protection against MenACWY IMD was 78.9% in infants and 74.1% in toddlers. The model also assumed that cross-protection only applied in vaccinated individuals fully protected against MenB, thus the overall cross-protection effectiveness of 4CMenB against MenACWY IMD was 62.9% in infants and toddlers.

## Results

**Figure 4.**
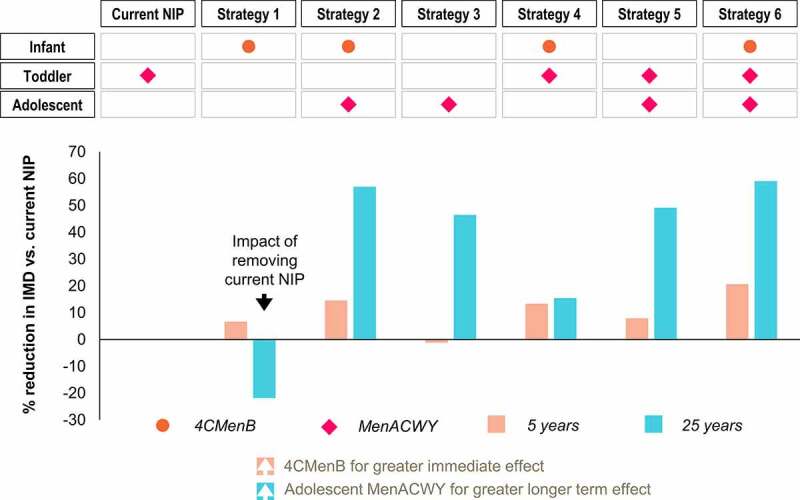
Percent reduction in IMD incidence after 5 years and 25 years – impact of introducing each strategy versus the current NIP. Percent reduction in IMD incidence with each vaccination strategy compared with the National Immunization Program (NIP) after 5 years and 25 years of vaccination. Abbreviations: IMD, invasive meningococcal disease; MenACWY, quadrivalent meningococcal conjugate vaccine; 4CMenB, four-component meningococcal serogroup B vaccine.

The current NIP in Chile (MenACWY toddler dose at 12 months) provides direct protection against MenACWY IMD in the vaccinated population. The additional impact on the number of IMD cases of introducing each vaccination strategy compared with the current NIP in Chile is presented in Figure 4.

### Impact of targeting only infants/toddlers for vaccination: Strategies 1 and 4

Strategy 1 highlights the negative impact of removing the current MenACWY NIP: the resulting increase in MenACWY IMD counteracts the initial benefits (reduction in MenBWY IMD) from introducing infant 4CMenB (Figure 4).

Strategy 4 shows some benefits, after both 5 and 25 years, of adding infant 4CMenB to the current toddler MenACWY NIP, thereby directly protecting against MenABCWY IMD in the age group with the highest incidence and burden (Figure 4).

Strategies without 4CMenB (Strategies 3 and 5) do not achieve as great short-term benefits after 5 years (Figure 4).

### Impact of replacing toddler MenACWY with adolescent MenACWY: Strategies 2 and 3

Strategy 3 shows that replacing toddler MenACWY with adolescent MenACWY has a positive effect on IMD incidence after 25 years, with little effect after 5 years. Even greater 5-year and 25-year reductions are seen, however, when combining infant 4CMenB and adolescent MenACWY (Strategy 2) (Figure 4).

### Impact of targeting both infants/toddlers and adolescents for vaccination: Strategies 2, 5, and 6

These three strategies provide the greatest benefits, after 5 and 25 years. Infant 4CMenB with adolescent MenACWY (Strategy 2) prevents more IMD cases than toddler MenACWY with adolescent MenACWY (Strategy 5). Strategy 6 has the biggest reduction in IMD by combining infant 4CMenB with toddler and adolescent MenACWY (Figure 4).

### Largest vaccine impact in age group 0–4 years old

**Figure 5.**
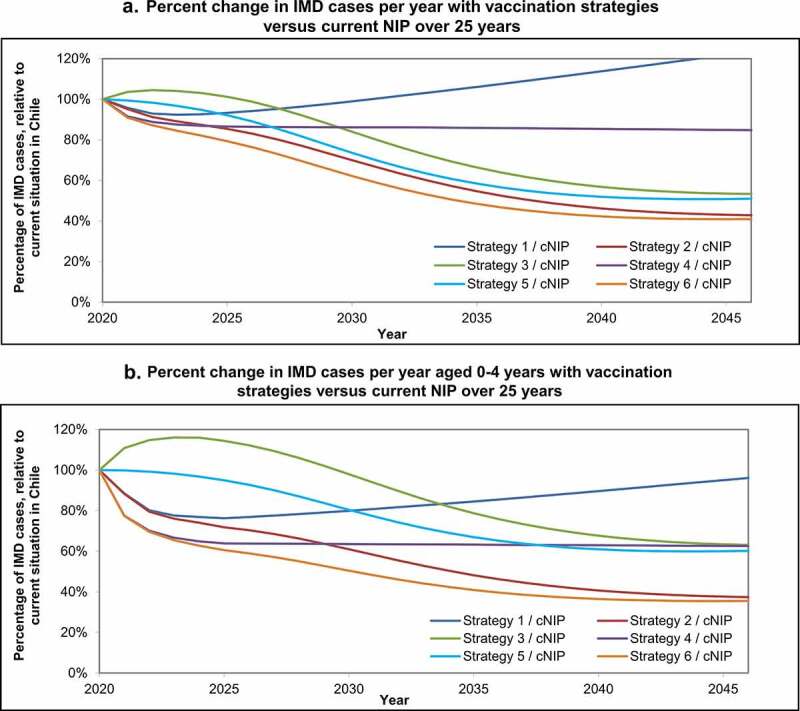
Percent change in IMD cases per year with vaccination strategies versus current NIP over 25 years, (a) for all ages and (b) for ages 0–4 years old. Percent change in invasive meningococcal disease (IMD) cases with each strategy compared with the current National Immunization Program (cNIP) over 25 years (a) for all ages, and (b) for ages 0–4 years.

Figure 5a shows the yearly percent change in IMD cases with each strategy, relative to the current NIP over 25 years. The greatest impact of these vaccination strategies is seen in the age group 0–4 years (infants and young children) who have the highest incidence, where there is a short-term direct effect of infant/toddler programs, and a long-term indirect herd protection effect from adolescent programs (Figure 5b). For example, in Figure 5b, having no toddler or infant vaccination and only an adolescent MenACWY program (Strategy 3) is less effective than the current toddler NIP in the short term, but more effective in the long term due to adolescent MenACWY vaccination herd effects that occur over time protecting other age groups.

**Figure 6.**
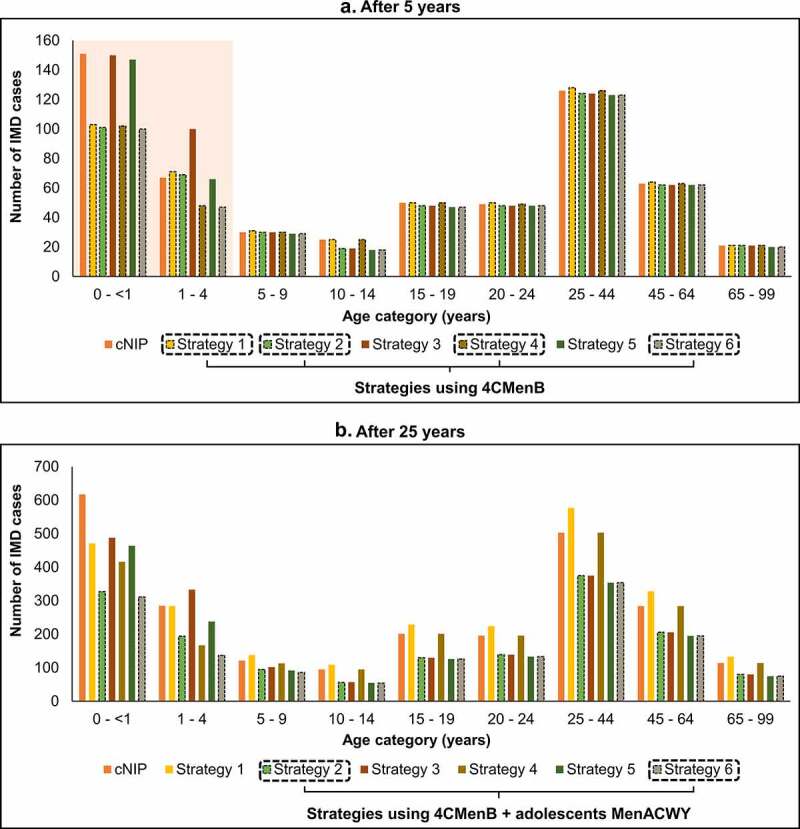
Impact of vaccination strategies: cumulative IMD cases by age group, (a) after 5 years and (b) after 25 years. Impact of vaccination strategies on the cumulative number of invasive meningococcal disease (IMD) cases by age group compared with the current National Immunization Program (cNIP), (a) after 5 years of vaccination, and (b) after 25 years of vaccination. Abbreviations: 4CMenB, four-component meningococcal serogroup B vaccine; MenACWY, quadrivalent meningococcal conjugate vaccine.

Therefore, the addition of 4CMenB to any of the programs is a key driver of the short-term impact and adds to the long-term impact as well. Introducing adolescent MenACWY, instead of or in addition to an infant/toddler program, is a key driver of long-term impact. The impact of adding 4CMenB strategies is especially evident in infants aged <1 year and young children aged 1–4 years in the short term (Figure 6a). Strategies combining infant 4CMenB and adolescent MenACWY have the greatest impact across all age groups, especially seen in the longer term (Figure 6b).

### Impact on sequelae, deaths, and QALYs

**Figure 7.**
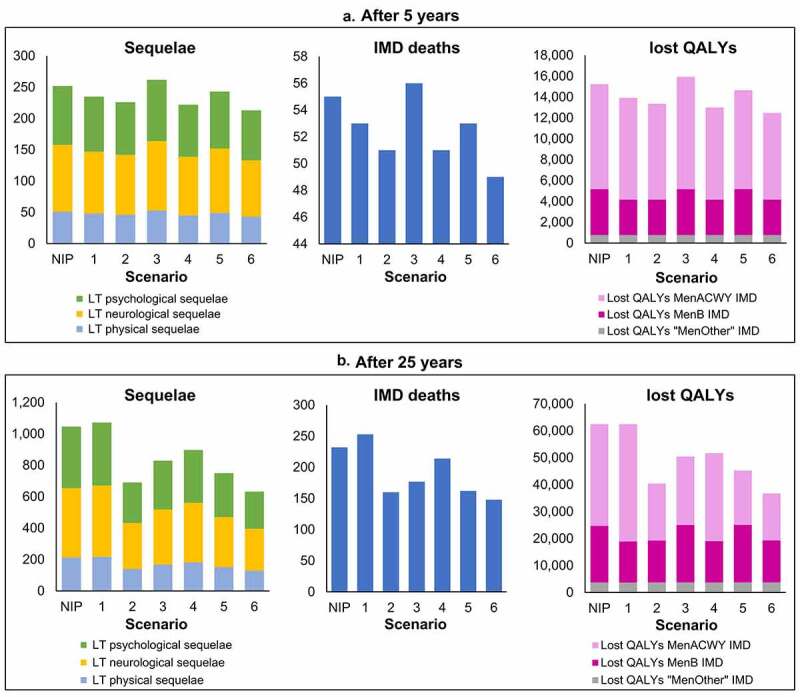
Impact on long-term sequelae, IMD deaths and lost QALYs due to IMD with each strategy versus the current NIP, after (a) 5 years and (b) 25 years. The impact of each vaccination strategy (1–6) compared with the National Immunization Program (NIP) in Chile on invasive meningococcal disease (IMD)-related long-term (LT) physical, neurological, and psychological/behavioral sequelae; IMD deaths; and lost quality-adjusted life-years (QALYs) due to MenACWY, MenB, or “MenOther” IMD is presented after 5 years and after 25 years of vaccination.

Apart from Strategy 1, which resulted in a lower reduction in IMD cases compared with the current NIP, all other strategies resulted in greater reductions in IMD incidence and therefore, a reduced occurrence of IMD-related sequelae, deaths, and lost QALYs. Figure 7 shows the impact on health outcomes of each strategy versus the current NIP after 5 years (Figure7a) and 25 years (Figure 7b).

## Discussion

Following the serogroup W emergence and outbreak, affecting mainly young children in 2012, MenACWY vaccination was introduced for toddlers and has been successful in protecting this age group. Infants and older age groups continue to be at risk from serogroup W IMD and could benefit from adolescent vaccination to induce herd immunity in the whole population. Young children remain at risk from serogroup B IMD. This study explored the impact of six alternative vaccination strategies to control IMD in Chile and reduce the burden in the wider population.

Two vaccination strategies had the greatest public health impact (Strategies 2 and 6), resulting in the highest reduction in IMD in all age groups and further reducing IMD-related long-term sequelae, deaths, and QALYs lost compared with the current NIP. Strategy 6 combined infant 4CMenB (to reduce MenB IMD and, through cross-protection, provide some protection in the short term against MenWY IMD) with toddler MenACWY vaccination (protecting young children against MenACWY IMD in the short term) and with adolescent MenACWY vaccination (to directly protect adolescents and over time indirectly protect young children and older age groups through herd immunity). Strategy 2 combined infant 4CMenB with adolescent MenACWY vaccination, thereby protecting infants against MenB IMD and with cross-protection against MenWY IMD in the short term until herd protection effects from adolescent MenACWY vaccination are established.

In Strategies 1–3, the impact of introducing infant 4CMenB and/or adolescent MenACWY vaccination instead of the current toddler MenACWY NIP was assessed. Given the epidemiology of IMD at the time of the outbreak and the rising predominance of MenW, the results suggest that only introducing infant 4CMenB (Strategy 1) was less effective than the current NIP in controlling IMD, whereas only introducing adolescent MenACWY (Strategy 3) was more effective than the current NIP in the long term, due to herd protection effects that occur with time, and introducing infant 4CMenB with adolescent MenACWY (Strategy 2) produced more public health benefits compared with the current NIP.

The impact of 4CMenB in the model could be underestimated, as a conservative estimate for 4CMenB cross-protection was used (assuming no cross-protection against serogroup C IMD). As shown in Figure 2, MenB and MenW are currently the main causes of IMD in Chile, and 4CMenB vaccination has the potential to prevent both. The model assumed a conservative cross-protection effectiveness of 62.9% with infant and toddler 4CMenB vaccination. Real-world evidence in the first 3 years following routine infant 4CMenB vaccination in the UK suggests that MenW IMD decreased by 69% in vaccine-eligible age groups, regardless of vaccination status.^15^

Compared with the current NIP, Strategy 4 (adding infant 4CMenB to the current NIP) produces a greater reduction in IMD in the short term, and Strategy 5 (adding adolescent MenACWY to the current NIP) produces a greater reduction in IMD in the long term.

The epidemiology of meningococcal disease is unpredictable, and the public health consequences of an outbreak are severe, as witnessed in Chile in 2012. A systematic review in Latin American countries reported large fluctuations in IMD incidence as well as CFRs within and across countries. While MenB (29% in 2012) and MenC (44% in 2012) were responsible for most IMD cases, MenW was also increasingly reported in Argentina, Brazil, and Uruguay. Studies on carriage of *Neisseria meningitidis* frequently found that MenB and MenC were the most prevalent.^2^ This highlights the need to broaden protection against all serogroups causing IMD.

The importance of increasing the impact of existing vaccination programs by considering herd effects was highlighted by the 2015 Global Meningococcal Initiative. Vaccination strategies with a high uptake in age groups with the highest carriage, i.e., adolescents, can play an important role in protecting the wider population by reducing transmission.^1^ Carriage prevalence differs by geographic region but is generally found to be highest in adolescents. In 2017, Argentina also introduced MenACWY into its NIP in response to the increasing burden of serogroup W IMD from 2012 onwards. The NIP in Argentina included infant vaccination (at 3, 5, and 15 months) as well as a single dose in adolescents designed to reduce carriage and therefore induce herd immunity.^47^

In 2013, a large cross-sectional study in Chile found an overall carriage rate of 6.5% (95% confidence interval [CI] 5.7–7.3) among 10- to 19-year-olds, with the highest prevalence among 14- to 17-year-olds of 7.6% (95% CI 6.5–8.8).^24^ Although these carriage rates are lower than those reported in countries in Europe, similar carriage rates of 4.9% (95% CI 3.6–6.1) were reported in adolescents in Brazil^48^ and a recent systematic review including countries in Latin America reported carriage rates ranging from 1.6% to 9.9%.^47^ Variability in IMD epidemiology data is likely to be due to limitations with surveillance, reporting and notification of IMD, leading to under-reporting and underestimation of IMD in the region,^2,47,49^ although in Chile and Brazil, vast improvements in surveillance and diagnosis of IMD were noted.^50^

In some countries, there has been a link between increasing carriage (of serogroup B or X) and an increase in cases.^39^ Two studies in Chile observed a low prevalence of serogroup W in adolescent carriers, despite this serogroup causing the majority of IMD cases in the same year (2012).^24,39^ Diaz et al.^24^ suggest that the duration of carriage with hypervirulent strains like W-135 may in fact be shorter and therefore not as frequently observed in carriage studies. Rubilar et al.^46^ suggest that the lack of an observed increase in serogroup W carriage to match the increase in serogroup W IMD cases may be explained by the increased virulence of the strain that caused the epidemic in Chile.^39^ The observed discrepancy between serogroup W prevalence rates in carriers and those in IMD cases highlights the difficulties in predicting the epidemiology of IMD based on carriage rates. Despite the low prevalence of serogroup W in carriage studies, they allowed to identify the hypervirulent clone in circulation, which prompted the Ministry of Health’s action plan.^49^

Decision-analytic models with a dynamic transmission component provide a robust method to model infectious diseases and the potential impact of vaccination in the population. This DyCE model is unique in being able to model the impact of vaccines targeting MenACWY and MenB IMD in various combinations. There were, however, some limitations. While every effort was made to include IMD and population data from Chile, data from other countries were used to estimate sequelae risk, carriage by age group, and utility loss, due to a lack of Chilean data. In addition, the incidence of IMD is unpredictable and assumptions were needed to estimate the projected incidence in the absence of vaccination following the outbreak in 2013.

In conclusion, the results of this analysis will help policymakers determine the best strategy to maximize the benefits of meningococcal vaccines targeting infants, young children, and adolescents. The current management of IMD in Chile could be improved by introducing routine infant 4CMenB, an effective measure to achieve even greater IMD reduction in infants with an immediate effect that is sustained over time, and adolescent MenACWY, which provides additional protection to all age groups over time. This combined strategy protects against IMD from serogroups A, B, C, W, and Y and has the potential to provide a positive public health impact in all age groups.

## Supplementary Material

Supplemental Material

## Trademark

*Bexsero* and *Menveo* are trademarks owned by or licensed to the GSK group of companies.
