# Supplementary material for: Modeling the public health impact of different meningococcal vaccination strategies with 4CMenB and MenACWY versus the current toddler MenACWY National Immunization Program in Chile
Source: Hum Vaccin Immunother. 2021 Dec 10;17(12):5603–13. doi: 10.1080/21645515.2021.1996808 (PMC8904027; doi:10.1080/21645515.2021.1996808)
Supplement: Supplemental Material [file KHVI_A_1996808_SM3082.docx]

**Appendix**

**Manuscript title:** Modeling the public health impact of different meningococcal vaccination strategies with 4CMenB and MenACWY versus the current toddler MenACWY National Immunization Program in Chile

**1. Case-fatality rate (CFR) by age group**

The mortality rates related to acute phase invasive meningococcal disease (IMD) were calculated by Dr Gabriel Cavada (Escuela de Salud Pública, Universidad de Chile, Santiago, Chile) using the number of cases and deaths obtained from the Hospital Discharge Database 2012–2018 from the health statistics department in Chile. The CFRs were estimated based on 61 deaths and 568 IMD cases registered. The resulting CFRs for each age group are presented in Appendix Table 1.

**Appendix Table 1.** CFRs for all serogroups.

| Age (years) | CFR by age group | 95% CI Lower bound | 95% CI Upper bound | Standard error |
| --- | --- | --- | --- | --- |
| 0–0y | 4.62% | 1.92% | 9.92% | 1.84% |
| 1–4y | 6.58% | 2.50% | 14.84% | 2.84% |
| 5–9y | 5.88% | 0.65% | 20.07% | 4.04% |
| 10–14y | 10.00% | 1.57% | 31.32% | 6.71% |
| 15–19y | 11.54% | 3.18% | 29.80% | 6.27% |
| 20–24y | 4.17% | 0.00% | 21.87% | 4.08% |
| 25–44y | 10.64% | 5.70% | 18.67% | 3.18% |
| 45–64y | 23.96% | 16.47% | 33.45% | 4.36% |
| 65–99y | 13.24% | 6.90% | 23.50% | 4.11% |

Abbreviations: CFR, case-fatality rate; CI, confidence interval; y, years.

**2. Probability and utility loss of long-term sequelae**

Surviving IMD patients may suffer from long-term sequelae that in many cases are life-long. The risk of developing each sequela assumed in the model is presented in Appendix Table 2. The probabilities and utilities associated with long-term sequelae are used for presenting the number of cases of long-term sequelae and calculating the quality-adjusted life-year (QALY) loss due to long-term sequelae. The calculations are made in the decision tree. Findings of observational studies in IMD survivors suggest multiple sequelae can occur.^1-3^ With very limited data available for the likelihood of co-occurrence of specific sequelae and limited age-stratified data for occurrence of sequelae, the decision tree part of the DyCE (Dynamic transmission-based Cost-Effectiveness) model assumes the probability of developing each sequela is independent (i.e., the probability of developing a particular sequela is assumed to be independent of whether or not the patient develops any of the other sequelae) and does not differ by age.

**Appendix Table 2.** Risk of developing long-term IMD sequelae (%) and health state utility.

| **Sequelae** | **Probability** | **Utility** |
| --- | --- | --- |
| ***Physical sequelae*** |  |  |
| Skin scarring | 6.39%^1^ | 1^4^ |
| Amputation with severe disability | 1.26%^3^ | 0.69^5^ |
| Renal dysfunction/failure/insufficiency | 2.05%^1^ | 0.82^6^ |
| ***Neurologic sequelae*** |  |  |
| Hearing loss: unilateral/hearing impairment | 5.21%^3^ | 0.91^7^ |
| Hearing loss: moderate bilateral | 3.80%^3^ | 0.91^7^ |
| Hearing loss: severe/profound bilateral/deafness (cochlear implant) | 2.45%^3^ | 0.81^7^ |
| Speech or communication problems | 3.56%^3^ | 0.78^8^ |
| Epilepsy/seizures | 1.78%^3^ | 0.83^7^ |
| Motor deficits | 1.53%^1^ | 0.67^7^ |
| Severe neurological disorders | 1.02%^1^ | 0.62^7^ |
| Mental retardation/low IQ | 0.50%^3^ | 0.62^7^ |
| Blindness/severe visual impairment | 0.42%^3^ | 0.26^9^ |
| ***Psychologic and behavioral sequelae*** |  |  |
| ADHD | 9.66%^3^ | 0.75^10^ |
| Separation anxiety | 5.96%^3^ | 0.75^11^ |
| Anxiety | 2.25%^3^ | 0.75^11^ |
| Depression | 0.00%^3^ | 0.789^11^ |

Abbreviations: ADHD, attention deficit hyperactivity disorder; IQ, intelligence quotient; IMD, invasive meningococcal disease.

**References**

1. Bettinger JA, Scheifele DW, Le Saux N, Halperin SA, Vaudry W, Tsang R, For the Members of Canadian Immunization Monitoring Program AI. The disease burden of invasive meningococcal serogroup B disease in Canada. Pediatr Infect Dis J 2013; 32:e20-e25. doi: 10.1097/INF.0b013e3182706b89 [doi]

2. Olbrich KJ, Muller D, Schumacher S, Beck E, Meszaros K, Koerber F. Systematic review of invasive meningococcal disease: sequelae and quality of life impact on patients and their caregivers. Infectious diseases and therapy 2018; 7:421-438. doi: 10.1007/s40121-018-0213-2

3. Viner RM, Booy R, Johnson H, Edmunds WJ, Hudson L, Bedford H, Kaczmarski E, Rajput K, Ramsay M, Christie D. Outcomes of invasive meningococcal serogroup B disease in children and adolescents (MOSAIC): a case-control study. Lancet Neurol 2012; 11:774-783. doi: S1474-4422(12)70180-1 [pii];10.1016/S1474-4422(12)70180-1 [doi]

4. Blakeney P, Meyer W, 3rd, Robert R, Desai M, Wolf S, Herndon D. Long-term psychosocial adaptation of children who survive burns involving 80% or greater total body surface area. J Trauma 1998; 44:625-632; discussion 633-624. doi: 10.1097/00005373-199804000-00011

5. Erickson LJ, De WP, McMahon J, Heim S. Complications of meningococcal disease in college students. Clinical infectious diseases : an official publication of the Infectious Diseases Society of America 2001; 33:737-739. doi: CID000945 [pii];10.1086/322587 [doi]

6. Wyld M, Morton RL, Hayen A, Howard K, Webster AC. A systematic review and meta-analysis of utility-based quality of life in chronic kidney disease treatments. PLoS medicine 2012; 9:e1001307. doi: 10.1371/journal.pmed.1001307

7. Oostenbrink R, HA AM, Essink-Bot ML. The EQ-5D and the Health Utilities Index for permanent sequelae after meningitis: a head-to-head comparison. J Clin Epidemiol 2002; 55:791-799. doi:

8. Al-Janabi H, Van Exel J, Brouwer W, Trotter C, Glennie L, Hannigan L, Coast J. Measuring Health Spillovers for Economic Evaluation: A Case Study in Meningitis. Health economics 2016; 25:1529-1544. doi: 10.1002/hec.3259

9. Brown MM, Brown GC, Sharma S, Kistler J, Brown H. Utility values associated with blindness in an adult population. The British journal of ophthalmology 2001; 85:327-331. doi:

10. Bennett JE, Sumner W, 2nd, Downs SM, Jaffe DM. Parents' utilities for outcomes of occult bacteremia. Arch Pediatr Adolesc Med 2000; 154:43-48. doi:

11. Saarni SI, Suvisaari J, Sintonen H, Pirkola S, Koskinen S, Aromaa A, Lonnqvist J. Impact of psychiatric disorders on health-related quality of life: general population survey. Br J Psychiatry 2007; 190:326-332. doi: 10.1192/bjp.bp.106.025106
